# The Polymorphism of the ACE Gene Affects Left Ventricular Hypertrophy and Causes Disturbances in Left Ventricular Systolic/Diastolic Function in Patients with Autosomal Dominant Polycystic Kidney Disease 

**DOI:** 10.1155/2014/707658

**Published:** 2014-01-02

**Authors:** Maria Wanic-Kossowska, Bartlomiej Posnik, Mikolaj Kobelski, Elzbieta Pawliczak, Krzysztof Pawlaczyk, Krzysztof Hoppe, Krzysztof Schwermer, Dorota Sikorska

**Affiliations:** Department of Nephrology, Transplantology and Internal Medicine, Poznan University of Medical Sciences, 60-355 Poznan, Poland

## Abstract

Autosomal dominant polycystic kidney disease (ADPKD) is one of the most frequently occurring autosomal diseases inherited in the dominant manner. Due to this, lesions in the cardiovascular system of ADPKD patients have caught the attention of clinical investigators worldwide. The aim of the study was to analyse cardiovascular complications in ADPKD patients with a focus on left ventricular hypertrophy (LVH) and selected components of its systolic/diastolic function based on echocardiography. The study was conducted on 55 patients with ADPKD (24 males, 31 females), subdivided into three groups according to the stage of chronic kidney disease (CKD). The patient group with ADPKD and ESRD (group C) manifested an increased incidence of the D allele as compared to group A and group B (*χ* ^2^ = 4.217, *P* = 0.04). In all ADPKD patients with the DD genotype, left ventricular mass (LVM), posterior wall thickness (PWT), and interventricular septal thickness (IVS) were significantly higher compared to patients possessing the II and ID genotypes (*P* < 0.02, *P* < 0.003, and *P* < 0.009, resp.). The DD genotype exists more frequently in ADPKD patients with ESRD and is associated with a higher occurrence of LVH and disturbances in systolic-diastolic function when compared to ADPKD ESRD patients with the II and ID genotypes.

## 1. Introduction

Autosomal dominant polycystic kidney disease is one of the most common inherited kidney diseases, with an incidence estimated at 1 : 1000 examined individuals. Patients with ADPKD comprise about 8–10% of patients on renal replacement therapy [1]. Genetic and experimental studies on animal models, in tissue cultures, and clinical investigations have demonstrated that ADPKD manifests with the presence of multiple cysts, mostly in the kidneys (80–90%) and liver (50–90%), and less frequently in the pancreas (up to 10%), spleen (up to 5%), ovaries, uterus, central nervous system, and connective tissue structures [2]. Such a wide spectrum of multiorgan lesions suggests that ADPKD can be rightfully regarded as a systemic disease that may manifest at any time [3].

Lesions of the cardiovascular system in ADPKD patients have been an area of interest for clinical investigators; analysis of these lesions was first presented by Leier et al. [4] in 1984. Using retrospective and prospective analyses conducted on 62 ADPKD patients, the authors demonstrated lesions of the cardiovascular system in 18% of the cases. In 6 patients the lesions were verified using histopathology. Traits of myxomatous degeneration, fragmentation, and wasting of collagen were demonstrated within the heart valves in all 6 cases. The authors related these lesions to a generalized connective tissue dysfunction, manifested by a deficiency or complete absence of type III collagen. Similar lesions in cardiac valves of ADPKD patients were described by several authors, all of whom hypothesized that the observed disturbances were linked to a defect in the extracellular matrix identical to that which composes the epithelial matrix of renal tubules [4–8].

Apart from cardiac valve lesions, structural and functional alterations of the myocardium were noted in young individuals with ADPKD in whom no arterial hypertension or signs of renal injury were detected. With the use of echocardiographic and scintigraphic analyses, there has been demonstrated a significantly higher left ventricular mass index in groups of young individuals with ADPKD as compared to control groups [9, 10]. In a study conducted by Saggar-Malik et al. [11], young individuals with ADPKD demonstrated a 23% higher left ventricular mass index as compared to healthy individuals. The presence of early myocardial lesions was confirmed by Bardají et al. [12], a study which demonstrated a significant thickening of the interventricular septum and left ventricular posterior wall apart from an increased index of left ventricular mass. This was found in a group of 46 persons with a mean age of 25 years, with normal arterial blood pressure, but burdened with polycystic kidney disease lacking traits of disturbed renal function. Interestingly, and for the first time in relevant studies, the authors also noted a decreased ability in relaxation and an abnormal diastolic compliance of the left ventricle [12].

In recent years, several reports have appeared related to insertional-deletional (I/D) polymorphisms of the angiotensin-converting enzyme gene I (ACE) and to the effects of individual genotypes on the development of arterial hypertension, kidney failure, and the incidence of cardiovascular complications in ADPKD patients [13–22]. Opinions regarding a correlation between the I/D polymorphism of the ACE gene and the course of ADPKD are divergent. Some authors claim that ADPKD patients representing homozygotes (DD) of the ACE deletion allele manifest a higher risk of kidney failure at a younger age [16]. A study by Persu et al. analyzed the ACE gene polymorphism and its course of disease in 191 patients with ADPKD [20]. In the entire studied sample of patients, the authors failed to demonstrate any relationship except for, in 97 men, the presence of the DD genotype that caused kidney failure development five years sooner [20]. In such patients, cardiovascular complications also proved to be more frequent. Reports are also available which deny that the ACE polymorphism has any effect on the course of ADPKD. Investigations by van Dijk et al. concluded that the ACE polymorphism exerted no effect on the course of ADPKD and, furthermore, that only the genetic type or presence of the PKD_1_ or PKD_2_ gene was significant [22].

The aim of this study was to analyse cardiovascular complications in ADPKD patients with a particular focus on LVH and selected components of its systolic/diastolic function based on echocardiographic investigations.

## 2. Material and Method

The studies were performed in the Department of Nephrology at the Poznan University of Medical Sciences in Poznan, Poland, in the Department of Biochemistry and Molecular Biology at the Pomeranian University of Medicine in Szczecin, Poland, and in the Day Ward of Cardiological Diagnosis, Poznan-Nowe Miasto, Poland. The study was fully licensed by the Regional Ethical Commission, and all patients expressed informed consent for participation. The studies were performed in 55 patients with autosomal dominant polycystic kidney disease (ADPKD), including 24 males and 31 females, with a mean age of 49.56 ± 8.56 years. The timeframe of the study and patient monitoring was 60 months. ADPKD was diagnosed on the base of kidney ultrasonography, in line with the Ravina classification [23]. Arterial hypertension was recognized in 33 patients.

Patients participating in the study were divided into three groups depending on the advancement of their kidney disease according to the National Kidney Foundation Kidney Disease Outcome Quality Initiative (KDOQI) (Table 1).

- Group A included 18 patients (4 males and 14 females, mean age of 32.1 ± 14.5 years) with no traits of a disturbed renal function or at the 1st or 2nd stage of CKD (GFR ≥ 60 mL/min/1.73 m^2^).
- Group B included 13 patients (5 males and 8 females, mean age of 53.5 ± 11.3 years) at the 3rd or 4th stage of CKD and not treated with hemodialysis (GFR 15–59 mL/min/1.73 m^2^).
- Group C included 24 patients (15 males and 9 females, mean age of 57.0 ± 9.5 years) with ESRD treated with hemodialysis.

All of the patients received a transthoracic echocardiographic examination (ECHO) according to guidelines [24, 25] and basic laboratory tests in which serum concentrations of urea, creatinine, uric acid, cholesterol, hematocrit, and hemoglobin were examined. Each patient underwent an ECHO, including 2-dimensional, Doppler, and tissue Doppler imaging. An experienced echocardiographer, blinded to other measurements and qualifications of the studied groups, then analyzed the ECHO results. Creatinine clearance was calculated using the Cockroft-Gault formula [26]. Serum concentrations of urea, creatinine, and uric acid and the examination of blood smears were compiled using commercially available diagnostic kits.

The control group consisted of 30 healthy volunteers (12 males and 18 females, mean age of 45.3 ± 11.6 years) in whom the same examinations and laboratory tests were performed.

In every patient with ESRD (group C), hemodialysis was conducted three times a week, at an average of 4-5 hours each, for at least 6 months. In the course of the observation, the procedures were always performed in the same conditions, on the same day, and at the same hour.

### 2.1. Genetic Analysis

In all 55 patients with ADPKD, an insertional-deletional polymorphism of the ACE gene was determined. DNA was isolated from peripheral blood leukocytes using DNA isolation kits from Epicentre Technologies, USA (Master Pure TM Genomic DNA Purification Kit, cat. no. MG71100). For the evaluation of the polymorphism, polymerase chain reaction (PCR) was used to amplify 40 ng/*μ*L DNA samples in the 20 *μ*L terminal volume, containing 4 pM of each primer pair, 2.5 mM of every deoxyribonucleotide triphosphate (dATP, dTTP, dGTP, and dCTP), 2 *μ*L of PCR buffer (final concentrations of 1.5 mM MgCl_2_, 10 mM TRIS pH 8.3, and 50 mM KCl), and 0.5 U of Taq polymerase (MBI Fermentas).

The sense (ACE-1) and antisense (ACE-2) nucleotide primers involved, respectively, the oligonucleotide pairs of 5′-GCCCTGCAGGTGTCTGCAGCAGT-3′ and 5′-GGATGGCTCTCCCCGCCTTGTCTC-3′ (TIB Molbiol). Using the primers, PCR products included DNA fragments: for allele D a fragment of 319 base pair length and for allele I a fragment of 597 base pair length (79). Due to the phenomenon of preferential amplification of shorter DNA fragments (allele D) and, thus, the risk of underrating the incidence of the ID genotype, DNA samples from individuals preliminarily defined as DD homozygotes were verified using another PCR reaction with the primer pair of 5′-TGGGACCACAGCGCCCGCCACTAC-3′ as the sense primer (ACE-3) and 5′-TCGCCAGCCCTCCCATGCCCATAA-3′ as the antisense primer (ACE-4). The ACE-3 and ACE-4 primers are specific for allele I (79). A PCR product with a length of 395 base pairs provided proof for the presence of the ID genotype, and the lack of such a product confirmed the presence of the DD genotype. Amplification was achieved in a Mastercycler gradient thermocycler (Eppendorf) in the following conditions: preliminary denaturation at a temperature of 94°C for 5 min, followed by 35 cycles of denaturation at the temperature of 94°C for 15 seconds, primer annealing for 30 seconds at the temperature of 58°C (for ACE-1, ACE-2 primers) and 64°C (for ACE-3 and ACE-4 primers), and chain elongation at the temperature of 72°C for 30 seconds. PCR was completed with an 8 min chain elongation at a temperature of 72°C. Final amplification products were separated by electrophoresis in a 2% agarose gel and stained with ethidium bromide. The results were recorded using photography of the gels in UV light. Distribution of genotypes and alleles dependent on the ACE gene insertional-deletional polymorphism was evaluated in all 55 examined patients, and they were compared to the respective distribution in 100 healthy individuals, matched in respect to gender and age.

### 2.2. Statistical Analysis

Results were calculated using arithmetic mean and standard deviation. The normal distribution of the variables was checked using the Shapiro-Wilk test [27–29]. Relationships between examined indices were evaluated using either Pearson's linear correlation coefficient (for samples with normal distribution) or Spearman's correlation coefficient (for samples with distinct distribution) [27–29]. The data sets were compared to each other employing either the Student's *t*-test for unlinked samples (for data with normal distribution) or the Mann-Whitney's test (for unlinked variables with distinct distribution). In the evaluation of the ACE gene insertional-deletional polymorphism, frequency of the genotype and allele distribution was compared between groups of ADPKD patients and healthy individuals using the *χ* ^2^ test. Correlations between clinical/biochemical variables and respective genotype, dependent on ACE gene I/D polymorphism, were evaluated using the ANOVA rank Kruskal-Wallis test [27–29]. The value of *P* < 0.05 was accepted as a threshold for statistical significance.

## 3. Results

The studied patients with ADPKD manifested left ventricular hypertrophy as follows: in group A the hypertrophy was noted in 7 patients (39%), in group B in 12 patients (92%), and in group C in 16 patients (67%). The anatomic parameters of the left ventricle in ADPKD patients are listed in Table 2. The systolic function of the left ventricle in all 18 patients of group A showed no deviations from values obtained in the control group, but in 12 patients of group B and in 16 patients of group C the systolic function was impaired when compared to the control group (Table 2). Parameters evaluating left ventricular diastolic function differed in all studied patients compared to the control group.

43% of patients with ADPKD (*n* = 24) have manifested lesions within cardiac structures:

- aneurysm of the intra-atrial septum (*n* = 2),
- atrial-septal defect (*n* = 1),
- ostium secundum atrial-septal defect (*n* = 1),
- thickened mitral and aortal valve leaflets with fine calcifications (*n* = 10),
- traces of fluid in the pericardial sac (*n* = 6),
- inverted position of the heart (*n* = 1),
- mitral valve prolapse (*n* = 3).

### 3.1. Results of Genetic Analysis

The distribution of genotypes determined using the ACE gene insertional-deletional polymorphism in the entire studied group of patients was as follows: 8 patients (14%) demonstrated the DD genotype, 15 patients (27%) the II genotype, and 32 patients (59%) the ID genotype.

Distribution of genotypes in the control group of 100 healthy individuals was as follows: 35 individuals (35%) carried DD genotype, 45 persons (45%) manifested ID genotype, and 20 persons (20%) carried II genotype. Distribution of genotypes among patients with ADPKD was significantly different than the distribution noted in healthy individuals (*χ* ^2^ test, *P* < 0.05).

In the individual groups of patients, the distribution of genotypes conditioned by the ACE gene insertional-deletional polymorphism was as follows (Table 3).

- In 18 patients with GFR ≥ 60 mL/min/1.73 m^2^ (group A), 1 female patient carried the DD genotype (5.5%), 6 patients the II genotype (33.0%), and the remaining 11 patients the ID genotype (61.5%).
- In 13 patients with a 3rd-4th stage of CKD (group B), 1 female patient carried the DD genotype (7.7%), 5 patients the II genotype (38.0%), and the remaining 7 patients the ID genotype (54.3%).
- In 24 patients with ESRD (group C), 6 patients carried the DD genotype (25%), 4 patients the II genotype (16%), and the remaining 14 patients the ID genotype (59%).

Patients with ADPKD and ESRD (group C) were found to have an increased frequency of the DD genotype as compared to patients from groups I and II (*χ* ^2^ = 4.217, *P* = 0.04).

In patients with the DD genotype, significantly higher PWT (cm) and IVS thickness (cm) (1.22 ± 0.18 versus 0.97 ± 0.19, *P* < 0.003, and 1.34 ± 0.21 versus 1.08 ± 0.21, *P* < 0.009, resp.) and a significantly higher left ventricle mass (g) (313.1 ± 116.2 versus 202.9 ± 103.8, *P* < 0.02) were noted, as compared to patients with the II or ID genotypes.

As compared to group C patients with the II or ID genotype, patients in the same group but homozygotic for DD demonstrated a significantly higher RWT value (0.50 ± 0.07 versus 0.47 ± 0.07, *P* < 0.007), higher LVM (g) (333.4 ± 127.7 versus 234.8 ± 129.5, *P* < 0.006), higher thicknesses of PWT (cm) (1.24 ± 0.19 versus 1.05 ± 0.20, *P* < 0.0001) and IVS (cm) (1.35 ± 0.25 versus 1.17 ± 0.22, *P* < 0.0001), shortened IVRT time (ms) (90.8 ± 9.2 versus 107.2 ± 18.7, *P* < 0.01), prolongation of the A wave (cm/s) (87.7 ± 7.4 versus 80.8 ± 10.2, *P* < 0.002), lower values of HCT (32.6 ± 3.2 versus 36.2 ± 3.2, *P* < 0.01), and Hb concentrations (g/dL) (10.7 ± 1.0 versus 11.7 ± 1.1, *P* < 0.02), as well as a significantly higher creatinine concentration (mg/dL) (8.9 ± 1.8 versus 7.9 ± 1.0, *P* < 0.03).

In patients from group C, the following correlations were shown: between patient's age and creatinine clearance *r* = −0.53 (*P* < 0.001), PWT thickness *r* = 0.44 (*P* < 0.05), IVS thickness *r* = 0.57 (*P* < 0.001), diastolic volume of left ventricle (LVESV) *r* = 0.42 (*P* < 0.05), RWT value *r* = 0.43 (*P* < 0.05), left ventricular mass (LVM) *r* = 0.43 (*P* < 0.05), and LVFS value *r* = −0.47 (*P* < 0.05); between LVEF and creatinine concentration *r* = −0.45 (*P* < 0.05); between IVRT values and creatinine clearance *r* = −0.51 (*P* < 0.001); between left ventricular mass (LVM) and urea concentration *r* = 0.42 (*P* < 0.05).

## 4. Discussion

Results of our study are consistent with observations by authors who demonstrated the presence of higher left ventricular mass in patients with ADPKD as compared to a healthy population [30, 31]. Normotensive patients with autosomal dominant polycystic kidney disease with well-preserved renal function have significantly increased carotid intimal-medial thickness and significantly decreased coronary flow velocity reserve compared with healthy subjects [32]. These findings suggest that, in patients with ADPKD, atherosclerosis starts at an early stage in the course of the disease [32]. In the group of dialyzed patients with ADPKD, left ventricular mass has been documented to be significantly higher than in the control group. In group C, even in the 6 patients free of arterial hypertension, the left ventricular mass was significantly increased.

Among patients with ADPKD in the 3rd and 4th stage of CKD (Group B), left ventricular mass was significantly higher in 12 patients (92%), independently of their arterial blood pressure values. On the other hand, in patients without impaired renal function, the left ventricular mass was increased only in patients with arterial hypertension.

The correlations demonstrated in the group of patients with ESRD, between parameters of hypertrophy and systolic/diastolic function of the left ventricle on one hand and parameters of renal efficiency, indicate that increasing severity of renal disease is accompanied by an increasing mass and a deteriorating function of the left ventricle.

The presence of left ventricular hypertrophy promotes development of a systolic/diastolic cardiac insufficiency. Kunz et al. [33] and others [34] have demonstrated that, in 50–60% of patients with advanced kidney disease, a diastolic insufficiency of the left ventricle accompanies its hypertrophy. In a concentric hypertrophy of the left ventricle, the systolic function remains normal while impaired diastolic function represents the earliest sign of cardiac injury. Systolic failure develops a great deal less frequently and is often accompanied by left ventricular distension. In the diagnosis of diastolic dysfunction, an echocardiographic examination is of utmost importance because it may demonstrate early-restricted left ventricle diastolic filling with normal systolic function. The earlier affected diastolic function is likely due to the fact that diastole requires more energy for the dissociation of calcium from contractile proteins and for calcium's transport against a concentration gradient to the sarcoplasmic reticulum.

The observed decreased compliance of the ventricular wall in all studied patients with ADPKD may reflect an increased thickness of the walls, stromal fibrosis, or an increased collagen content. Decreased diastolic filling of the left ventricle leads to an increase in end-diastolic pressure and, subsequently, to an increased blood pressure in the pulmonary blood vessels. The cardiac lesions may explain the frequent complaints of exertional dyspnea noted in many patients. In our study, 80% of patients demonstrated concentric left ventricular hypertrophy and 20% demonstrated eccentric hypertrophy. The anatomical lesions have been accompanied by abnormal indices of left ventricular systolic/diastolic function. In all studied patients with ADPKD, increased end-systolic and end-diastolic left ventricular volumes were recorded. Analysis of the rate of blood flow through the mitral valve displayed a decrease in early and an increase in late mitral inflow, a lower ratio of the early to the late left ventricular filling, and an extended duration of isovolumetric diastole that may point to an extended left ventricular relaxation time.

Similar observations have been made by Oflaz et al. [35] during evaluation of left and right ventricular diastolic function in patients with ADPKD. It seems that, independently of their arterial blood pressure, patients manifested left ventricular hypertrophy with normal systolic function of the left ventricle, but diastolic function of both left and right ventricles was disturbed. The authors confirmed the already accepted hypothesis that hypertrophy of the left ventricle and disturbances in its function lead to a stimulation of the renin-angiotensin system, adrenergic stimulation, and hyperinsulinemia.

Turkmen at al. have demonstrated that carotid intimal and medial thickness was significantly increased, and coronary flow velocity reserve was significantly decreased in patients with ADPKD when compared with healthy subjects [36]. Ritz et al. [37] evaluated the degree of left ventricular hypertrophy in patients with ADPKD and in patients with chronic glomerulonephritis. All the patients represented a benign phase of renal disease. Upon autopsy, the left ventricular mass, expressed in percent of body weight, amounted to 0.706 in patients with ADPKD versus 0.661 in patients with glomerulonephritis. The authors stressed that the left ventricular mass was higher in patients with ADPKD who also carried valve defects. The presence of aortal-mitral defects was noted in 17% of patients with ADPKD but in only 5% of patients with glomerulonephritis. No differences could be disclosed in regard to the incidence of arteriosclerotic lesions present in coronary blood vessels. Third and fourth degree arteriosclerotic lesions were present in 67% of patients with ADPKD and in 61% of patients with glomerulonephritis.

The study performed in our department has led to similar observations; as many as 24 patients with ADPKD (43%) have manifested lesions within cardiac structures. Two patients have manifested an aneurysm of the intra-atrial septum, 1 patient has manifested an atrial-septal defect, 1 patient has manifested an ostium secundum atrial-septal defect, 10 patients have been observed having thickened mitral and aortal valve leaflets with fine calcifications, 6 patients were found with traces of fluid in the pericardial sac, 1 patient has shown an inverted position of the heart, and mitral valve prolapse was noted in 3 patients.

In recent years, several reports have pointed to the role of insertion-deletion polymorphisms within the gene coding for angiotensin convertase (ACE). Results have been inconsistent, with only some authors proving the effect of the ACE DD genotype on the progression of renal insufficiency and a higher incidence of cardiovascular complications [14, 16, 20]. However, there are also available data from other studies demonstrating the effects of the ACE polymorphism on the course of ADPKD that found no relationship between such a polymorphism and a higher mortality due to cardiovascular complications [13, 17, 22].

Among patients examined in this study, the frequency of the DD genotype was 14% (8 patients), II genotype 27% (15 patients), and the ID genotype 59% (32 patients). Among dialyzed ADPKD patients, DD genotype was in 6 (25%), II genotype in 4 (16%), and the ID genotype in 14 patients (59%). Similar observations were made in a study by Uemaso et al. [15], which analysed the frequency of individual alleles in dialyzed patients with ADPKD.

Results of our studies have unequivocally demonstrated that patients with ESRD, the last stage of CKD, manifest the DD genotype more frequently than ADPKD patients in the first four stages of CKD. Patients with ADPKD and the DD genotype have been recognized to manifest a significant left ventricular hypertrophy more frequently than the patients with the II or ID genotypes. Analysis of the three groups of patients has shown that, in patients with ESRD (group C), manifestation of the DD genotype is linked to a significant left ventricular hypertrophy and its disturbed diastolic function, as compared to patients in groups A and B.

Lesions in cardiac valves, leading to calcifications and functional insufficiency, have been noted in 10 dialyzed patients, 6 of whom carried the DD genotype. The noted lesions have been linked, in part, to the advancement of ADPKD. In one female patient with ADPKD having the DD genotype, but free of signs or symptoms of severe renal disease, was found an inverted position of the heart and other viscera (situs inversus). No patients with a persistent foramen ovale, aneurysm of the interventricular septum, or flaccid interventricular septum were found among those having the DD genotype. Uemaso et al. [15] made similar observations, noting no differences in the incidence of cardiac valve defects between patients with the DD genotype and patients with the II or ID genotype.

Results of our study confirmed opinions of other authors, who suggested an influence of the DD genotype on the incidence of cardiovascular complications among ADPKD patients [20]. Our results proved that, in men with ADPKD, the DD genotype was accompanied by an accelerated progression in kidney disease, as compared to patients with the II or ID genotype. In the presence of the DD allele, kidney failure was found to develop, on the average, 5 years earlier than in the presence of the II or ID allele. Baboolal et al. [16] demonstrated that a polymorphism of the angiotensinogen gene failed to affect renal function in patients with ADPKD. However, by examining effects of a polymorphism of the ACE gene, these study results noted that patients with the DD genotype manifested a higher risk of ESRD and an earlier requirement for dialysis therapy, before age 40, as compared to patients with the II genotype. Our study compared the duration in which ESRD developed between patients with the DD and those with the II genotype and found that, in homozygotes, signs of ESRD developed 7 years earlier. Similar observations were made in a study by Perez-Oller et al. [14] in which 151 patients with ADPKD, 32% with the DD genotype, started dialysis therapy before the age of 50. Studies by van Dijk et al. [22] and Saggar-Malik et al. [38] examined the effects of a polymorphism in the angiotensin and ACE genes on renal function in ADPKD patients and demonstrated no influence of such polymorphisms on creatinine level, creatinine clearance, incidence of cardiovascular complications, development of arterial hypertension, nor duration in which the patients developed renal failure.

## 5. Conclusions

In our study of patients with ADPKD and CKD, observed parameters of left ventricular hypertrophy, its systolic/diastolic function, and parameters of renal function may indirectly indicate that progression of the disease is linked to an increased left ventricular mass and its decreased function. Independently of the stage of CKD, in patients with ADPKD, diastolic function of the left ventricle becomes impaired while its systolic function remains within normal limits. The DD genotype is observed most frequently in patients with ADPKD and ESRD. As compared to patients with the II or ID genotypes, such patients show a significant left ventricular hypertrophy with a disturbed systolic/diastolic function.

In summary, cardiovascular complications, rapid development of renal failure, and arterial hypertension in patients with ADPKD are only partially dependent on the existing ACE genetic polymorphisms. In some cases, lesions in the cardiovascular and renal systems are induced or intensified by hormonal disturbances. Therefore, an early diagnosis and the implementation of nephroprotective and antihypertensive treatment may slow down CKD progression and decrease the risk of life-threatening complications.

## Figures and Tables

**Table 1:** Clinical data and laboratory results in 55 patients with ADPKD.

Parameter	Group A	Group B	Group C
No. of patients	18	13	24
Age (years)	32.1 ± 14.5	53.5 ± 11.3	57.0 ± 9.5
Urea (mg/dL)	29.7 ± 6.6	92.3 ± 36.2*	185.8 ± 29.1*
Creatinine (mg/dL)	1.0 ± 0.2	2.8 ± 1.8*	8.1 ± 1.3*
Creatinine clearance (mL/min)	106 ± 27	43 ± 21*	11 ± 3*
Uric acid (mg/dL)	5.3 ± 1.1	7.0 ± 1.7	7.7 ± 1.2
Hemoglobin (g/dL)	13.3 ± 1.3	13.5 ± 1.3	11.4 ± 1.1*
Hematocrit (%)	40.7 ± 3.1	41.1 ± 3.6	35.3 ± 3.5*

*P < 0.05 as compared to the control group.

**Table 2:** Echocardiography results in ADPKD patients.

Parameter	Control (*n* = 30)	Group A (*n* = 18)	Group B (*n* = 13)	Group C (*n* = 24)
LVM (g)	135.7 ± 31.3	148.9 ± 32.7	240.8 ± 94.0*	259.5 ± 133.6*
IVS (cm)	0.94 ± 0.13	0.94 ± 0.15	1.20 ± 0.20*	1.21 ± 0.23*
PWT (cm)	0.99 ± 0.16	0.85 ± 0.11	1.1 ± 0.2	1.10 ± 0.21*
RWT	0.41 ± 0.06	0.39 ± 0.05	0.47 ± 0.06*	0.49 ± 0.10*
LVEF (%)	62.2 ± 6.8	62.2 ± 8.4	54.2 ± 15.0	54.9 ± 11.2*
LVFS (%)	33.8 ± 4.9	36.5 ± 10.0	36.3 ± 11.2	36.9 ± 11.2
LVESV (mL/m^2^)	26.4 ± 11.7	37.6 ± 13.9*	49.7 ± 42.7*	57.6 ± 24.6*
LVEDV (mL/m^2^)	54.0 ± 15.7	98.6 ± 27.9*	108.1 ± 39.1*	134.4 ± 42.5*
*E* (cm/s)	88.5 ± 13.9	95.0 ± 23.2*	70.9 ± 18.2*	72.0 ± 25.4*
*A* (cm/s)	73.4 ± 10.5	62.2 ± 13.6*	82.9 ± 17.1*	82.5 ± 17.8*
*E*/*A*	1.24 ± 0.28	1.50 ± 0.49	0.80 ± 0.32*	0.80 ± 0.49*
IVRT (ms)	88.9 ± 13.9	92.0 ± 16.0	105.0 ± 26.0*	103.0 ± 18.0*

*P < 0.05.

**Table 3:** Distribution of genotypes related to insertion-deletion ACE gene polymorphism in 55 ADPKD patients.

I/D genotype of ACE	Group A	Group B	Group C
II	33.0%	38.0%	16.0%
ID	61.5%	54.3%	59.0%
DD	5.5%	7.7%	25.0%
